# Supplementary material for: Evidence for 4e charge of Cooper quartets in a biased multi-terminal graphene-based Josephson junction
Source: Nat Commun. 2022 May 31;13:3032. doi: 10.1038/s41467-022-30732-7 (PMC9156765; doi:10.1038/s41467-022-30732-7)
Supplement: Supplementary file 1 — Supplementary information [file 41467_2022_30732_MOESM1_ESM.docx]

SUPPLEMENTARY INFORMATION

**Evidence for 4*e* charge of Cooper quartets**

**in a biased multi-terminal graphene-based Josephson junction**

Ko-Fan Huang^1†^, Yuval Ronen^1†^, Régis Mélin^2^, Denis Feinberg^2^, Kenji Watanabe^3^, Takashi Taniguchi^4^, Philip Kim^1,5 *^

^1^ Department of Physics, Harvard University, Cambridge, Massachusetts 02138, USA

^2^ Université Grenoble – Alpes, CNRS, Grenoble INP, Institut NEEL, 38000 Grenoble, France

^3^ Research Center for Functional Materials, National Institute for Materials Science, 1-1 Namiki, Tsukuba 305-0044, Japan

^4^ International Center for Materials Nanoarchitectonics, National Institute for Materials Science, 1-1 Namiki, Tsukuba 305-0044, Japan

^5^ John A. Paulson School of Engineering and Applied Sciences, Harvard University, Cambridge, MA 02138, USA

^†^ These authors contributed equally to this work

* Corresponding author: [pkim@physics.harvard.edu](mailto:pkim@physics.harvard.edu)

**Section 0. Intrinsic synchronization for quartet transport: the context**

The multi-terminal Josephson junctions (MT-JJs) of this study pertain to the class of non-tunnel (metallic, quantum dot..) junctions where all properties derive from an Andreev bound states (ABS) spectrum. Experimentally, direct evidence for an ABS spectrum in a non-tunnel equilibrium JJ was obtained recently by side tunnel [1, 2, 3] and microwave [4, 5] spectroscopies. The current-phase relation (CPR) resulting from an ABS spectrum is generally anharmonic, $I_{J}(\varphi)=\underset{n}{\sum}I_{n}sin(n\varphi)$, contrarily to a tunnel junction where $I_{J}=I_{c}sin\varphi$. In the presence of a bias voltage V, the Josephson relation $\overset{\cdot}{\varphi}(t)=\frac{2e}{\hbar}V$ [6] implies a periodic sweeping in time of the ABS energies. While for a tunnel junction, one can safely write $I_{J}(t)=I_{c}sin\varphi(t)$, provided $eV<\Delta$, for a superconductor-normal metal-superconductor (SNS) junction this is not true anymore. Unless V is very small, nonadiabatic transitions mix the ABS and a new dynamical stationary state is obtained. As a consequence, multiple Andreev reflections (MAR) can promote a quasiparticle across the superconducting gap with the help of Cooper pairs gaining an energy $2eV$ [7, 8, 9]. This dissipative subgap transport is the hallmark of transparent SNS junctions. The interpretation of experiments with biased non-tunnel junctions relies on a full microscopic calculation using scattering theory [8] or nonequilibrium Green's functions (NEGF) [10]. To take into account the external circuit, an extension of the resistively-shunted junction (RSJ) model has been implemented by Chauvin et al. [11], at the expenses of injecting into the model the full-fledged $I(V)$ due to MARs, calculated with NEGF.

Quartet DC transport is a new phenomenon due to Andreev reflection (AR) in a non-tunnel MT-JJ. The formation of Cooper quartets in MT-JJs relies on crossed Andreev reflection [12-17] and Cooper pair splitting, which has been observed in several experiments with hybrid three-terminal junctions [18-24]. On the other hand, an extrinsic synchronization between two separate Josephson junctions was studied in the past in weak links [25, 26] coupled by an external impedance, showing plateaus in the $I(V)$ characteristics, thus a signature similar to that of quartets. Modelization of mode-locking stems from the classical RSJ model, featuring two coupled pendula. Mode-locking bears similarity with parametric locking on an external periodic field, that describes Shapiro steps in tunnel junctions. These models assume an adiabatic extension of the equilibrium CPR. Just as in two-terminal SNS junctions, this is not consistent in metallic MTJs, owing to the presence of AR and a complex ABS spectrum, extending to low energies. As pointed out in Ref. 27, quartets can be seen as a kind of ‘‘self-induced Shapiro steps’’. Nevertheless, those should be understood in the frame of metallic junctions with a complex ABS spectrum and MARs, not with RSJ models.

Similarly to Shapiro physics [6] but in non-tunnel junctions, we study the transport nonlinearities coming from the interplay between the MT-JJ ABS spectrum and the intrinsic phase dynamics. As in metallic JJs, a full microscopic NEGF calculation (see Section S5) provides features in qualitative agreement with the experiment. A simplified picture based on Floquet dynamics is also provided, that helps to understand the intrinsic resonances and the way they shift periodically with the applied magnetic flux. These new experimental features stem from the intrinsic synchronization responsible for the transport of the Cooper quartets, and they are distinct from the extrinsic synchronization of a mode-locking system.

The sections S1 -S4 of the Supplemental Material provide additional experimental information, and section S5 presents the theoretical modeling.

**Section 1. Dual voltage source for quartet measurement**

In order to control the potential of each superconducting terminal, the quartets are detected through the dual voltage source measurement scheme (Fig. Supplementary 1). The terminals $S_{1}$ and $S_{2}$ are biased with DC voltages $V_{b1}$ and $V_{b2}$ through a voltage divider and an RC filter (in the main text the voltage dividers are not shown). The loop terminal $S_{0}$ remains grounded at all times but on top of this DC ground, we apply a small AC excitation in the range of 0.25-0.3 V. Like the other leads, this lead also has a voltage divider followed by an RC filter. The voltage divider for the loop *S_0_* divides the AC excitation by ${10}^{5}$. In order to detect the quartet current, we measure the conductance at the biased terminals $S_{1}$ and $S_{2}$. As shown in the circuit, we use lock-in amplifier to measure the potential ${dV}_{1}$ and ${dV}_{2}$. The AC currents owing from are then given by $dI_{i}={dV}_{i}/r$, where $i=1,2$. Therefore, the conductance at each lead is $G_{i}=dI_{i}/dV={dV}_{i}/(r\cdot dV)$.


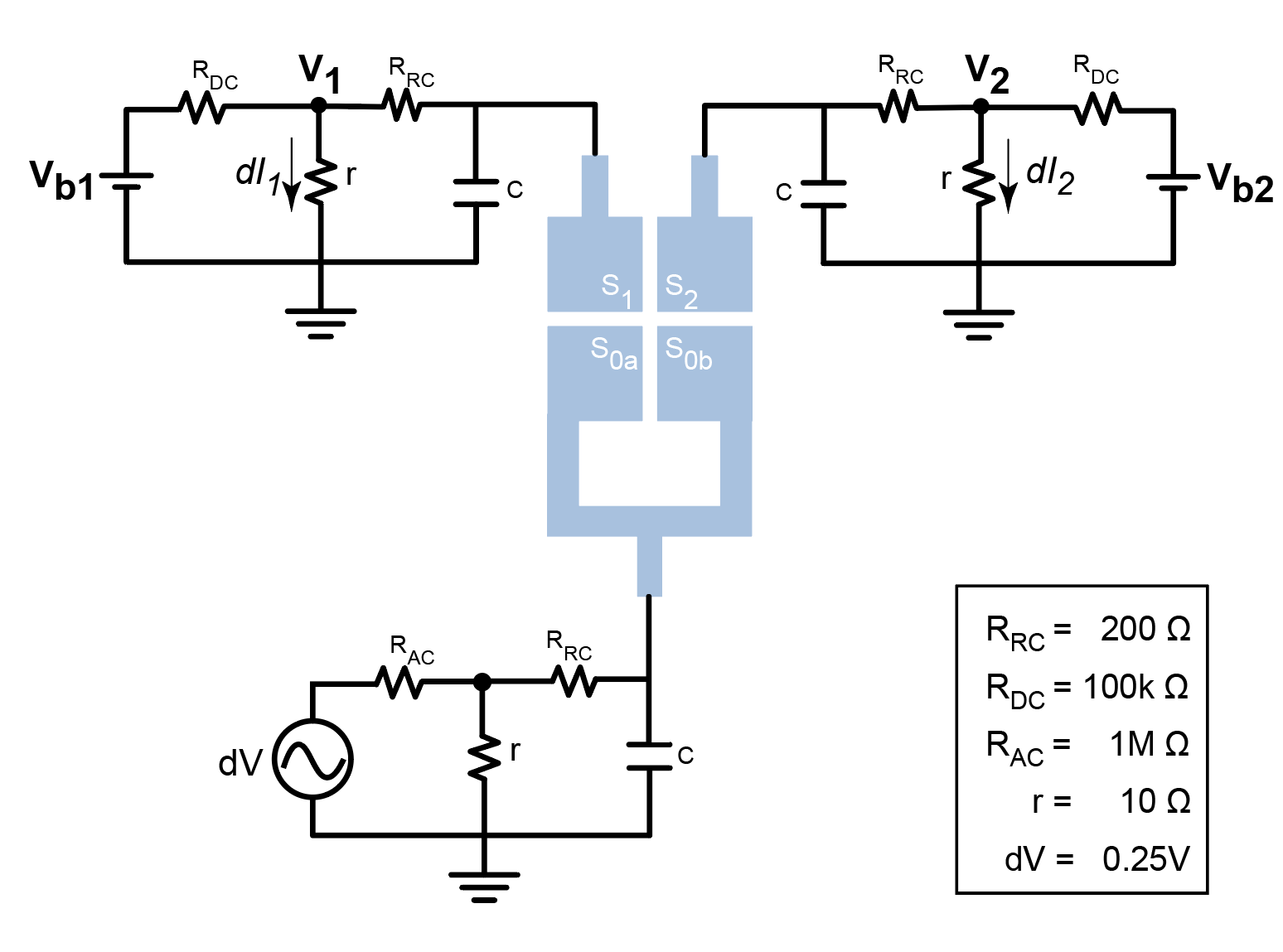


Fig. Supplementary 1: Schematic diagram of the dual voltage source for quartet measurement.

**Section 2. Comparison of currents at *S_1_* and *S_2_***

Figure Supplementary 2 shows the conductance $G_{i}=dI_{i}/dV$ measured at terminals $S_{1}$ and $S_{2}$, respectively. Similar features can be found in both, including the Josephson currents between any two leads and the quartet current along $V_{1}=-V_{2}$. Overall the conductance at $S_{2}$ is lower than that at $S_{1}$, suggesting the asymmetric couplings between each pair of contacts.


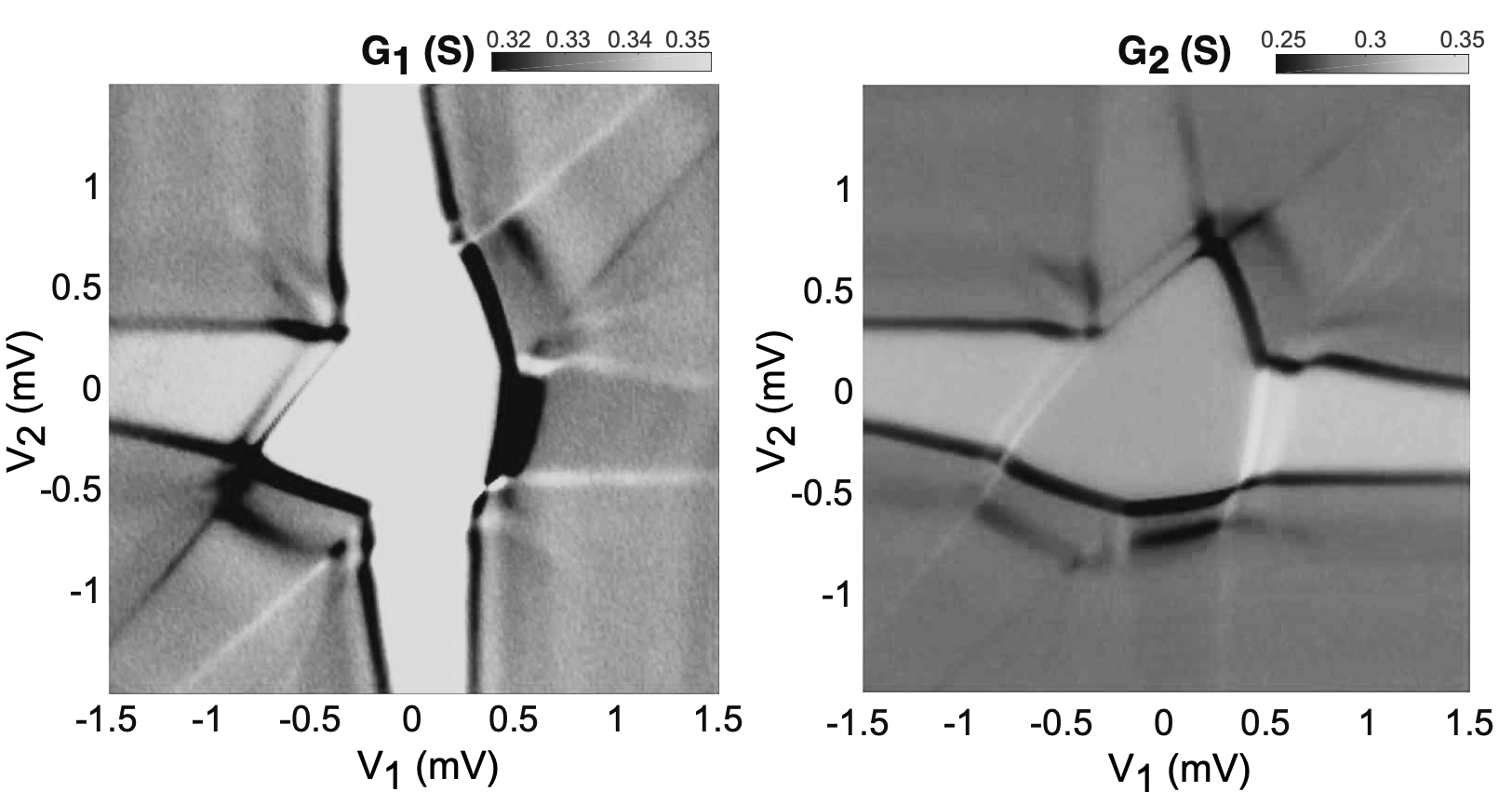


Fig. Supplementary 2: Comparative data between $G_{1}=dI_{1}/dV$ and $G_{2}=dI_{2}/dV$ as a function of $V_{1}$ and $V_{2}$.

**Section 3. Quartet on top of the background quasiparticle signal**

The quartet signal is much weaker than the Josephson signal. Yet it would not be observable in a tunnel junction, being a higher order process in the transmission, compared to a Josephson current. Its observation outside the zero-bias-voltage region, together with signals from quasi-particle background current in the background, is consistent with a quite transparent graphene junction. One should also add that MAR features might fade out at higher back-gate voltages due to an increase transmission [28, 29]. Figure Supplementary 3a shows the color plot of the conductance $G_{2}=dI_{2}/dV$ as a function of the bias $V_{1}$ and magnetic field while $V_{2}$ is fixed at 6 V. Figure Supplementary 3b shows the zoom-in scan around the quartet signal. The oscillations in magnetic field show a 3% variation of the total conductance (Fig. Supplementary 3c).


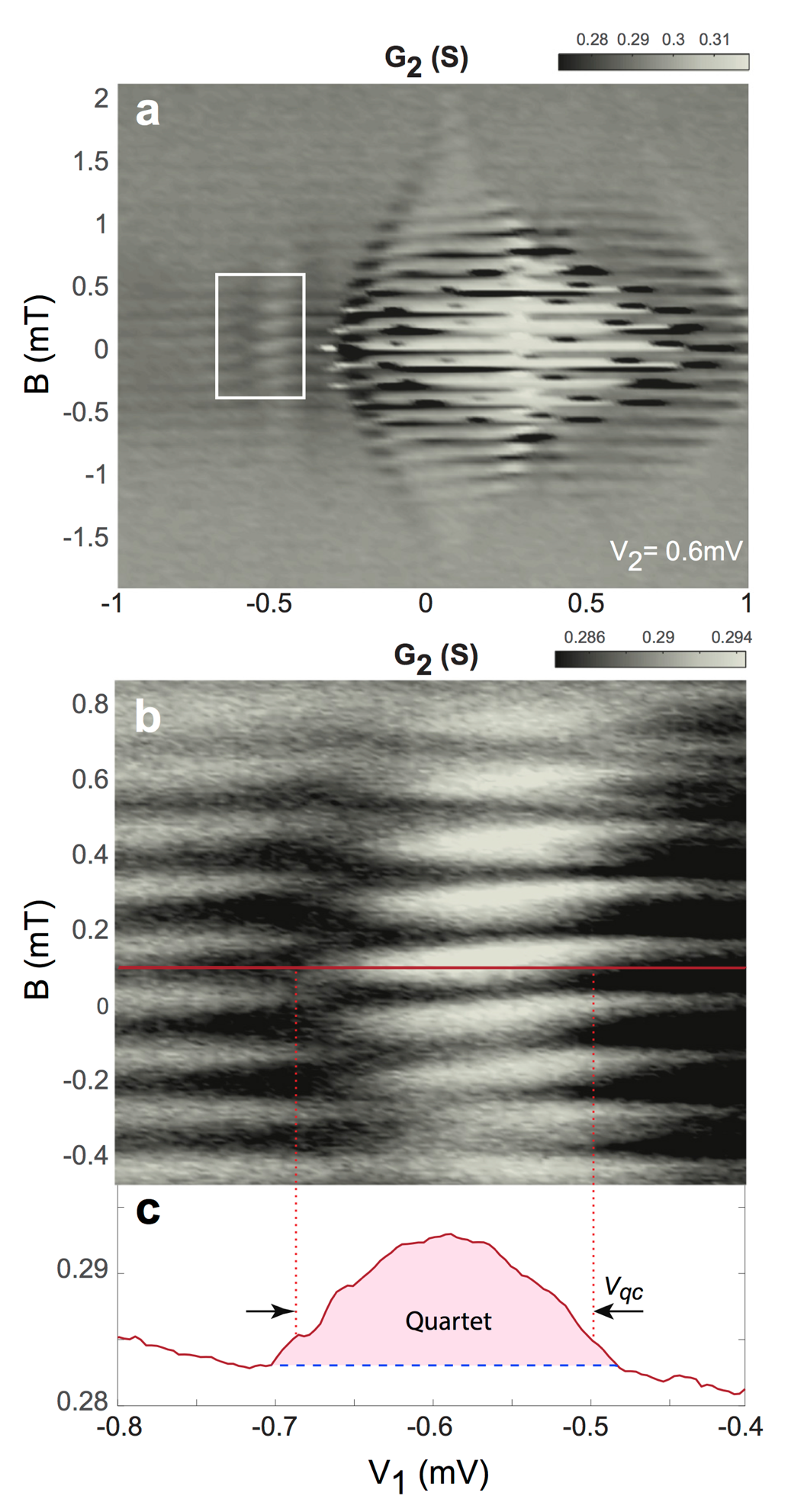


Fig. Supplementary 3: **a**. $G_{2}={dI_{2}}/{dV}$ as a function of the DC bias voltage $V_{1}$ and magnetic field when $V_{2}$ is fixed at 0.6 V with $V_{bg}$=40 V. The field-dependent quartet supercurrent is in the white box and the zoom-in scan is shown in **b**. Panel **c** shows the quartet signal on top of the quasi-particle background current (blue dashed line). $V_{qc}$ is the voltage corresponding to the quartet critical current $I_{qc}$.

**Section 4. Gate dependence of the multi-terminal graphene Josephson junction**

By applying a gate voltage, we can tune the chemical potential of the graphene channel region. Moreover, the gate voltage can change the density of states of the graphene underneath Al contacts, modulating the couplings between the graphene and the superconductors. Figure Supplementary 4 shows the differential conductance $G_{1}$, measured between $S_{1}$ and the grounded loop $S_{0}$, as a function of the two DC bias voltages $V_{1}$ and $V_{2}$ at back-gate $V_{bg}$= -10 V, -5 V, and 40 V. The critical value of each supercurrent is modulated accordingly, as well as the central zero-bias region. Note that among these back-gate voltages, the quartet supercurrent is the strongest at $V_{bg}$= -5 V.


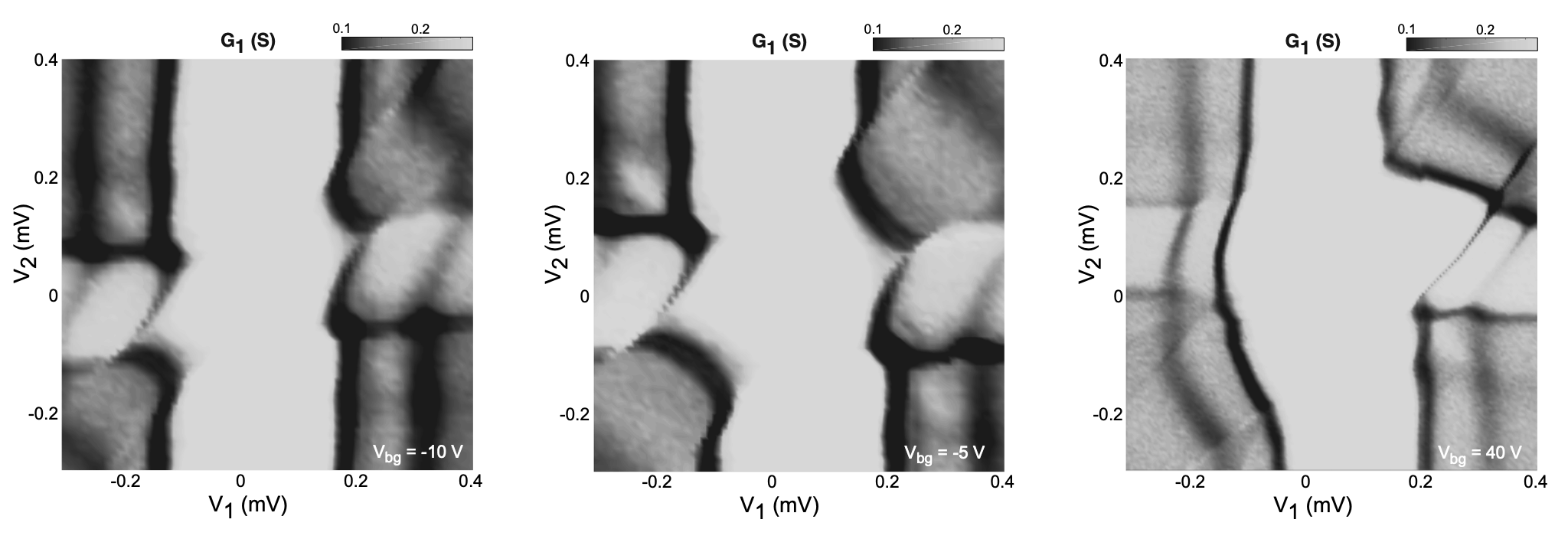


Fig. Supplementary 4: $G_{1}(V_{1}$, $V_{2})$ at $V_{bg}$ = -10 V, -5 V, and 40 V. Among these back-gate voltages, the quartet supercurrent (along $V_{1}=-V_{2}$) is the strongest at $V_{bg}$= -5 V.

**Secion 5. Theoretical complement for the interpretation of the experiment**

In this section, we discuss in detail the theoretical modeling. Subsection A presents a simple argument for why the quartet critical current can be larger at half flux quantum than in zero field, making reference to two recent theoretical papers [30, 31, 32]. Subsection B presents the basics of the Landau-Zener tunneling interpretation, starting with the simple example of a zero-dimensional quantum dot connected to four superconducting leads, described by the Hamiltonian in paragraph a, and its adiabatic and numerical solutions in paragraphs b and c. Paragraph d presents a mechanism for the inversion at finite bias voltage $V_{Q}$, driven by the Landau-Zener processes according to Ref. 30. Paragraph e extends the analysis to a two-channel model in order to solve some inconsistencies of the single-channel model, regarding the voltage range and the symmetry of the contact transparencies. This opens the perspective of future numerical studies of graphene quantum dots with arbitrary dimensions, connected to four superconducting leads and biased on the quartet line.

This work focuses on the conductance modulation along the quartet line, $V_{1}=-V_{2}=V$, due to an applied flux. Along this line, where the microscopic models are solved, the quartet phase $\varphi_{q}$ is stationary while the other conjugated phase variable $\varphi_{r}$ is time dependent. For a general JJ at a finite temperature, zero-bias conductance is finite and increases monotonically as the critical current increases [6]. In our multi-terminal JJ, a similar property holds along the quartet line, where the amplitude of the quartet conductance $G_{1}\left( V,-V \right)$ increases monotonically as the critical quartet current $I_{qc}\left( V,\Phi\right)$ increases [34]. Experimentally, this is shown in Fig. Supplementary 5. Therefore, the modulation of $G_{1}$ and $G_{2}$ as a function of the applied flux and on the applied quartet bias reflect the modulation of $I_{qc}\left( V,\Phi\right)$. The microscopic model presented in Refs 28, 29 provide the "quartet current"-"quartet phase" characteristics $I_{q}(\varphi_{q},V,\Phi)$ and one takes $I_{qc}(V,\Phi)=Max[I_{q}(\varphi_{q},V,\Phi]$ on $\varphi_{q}$.


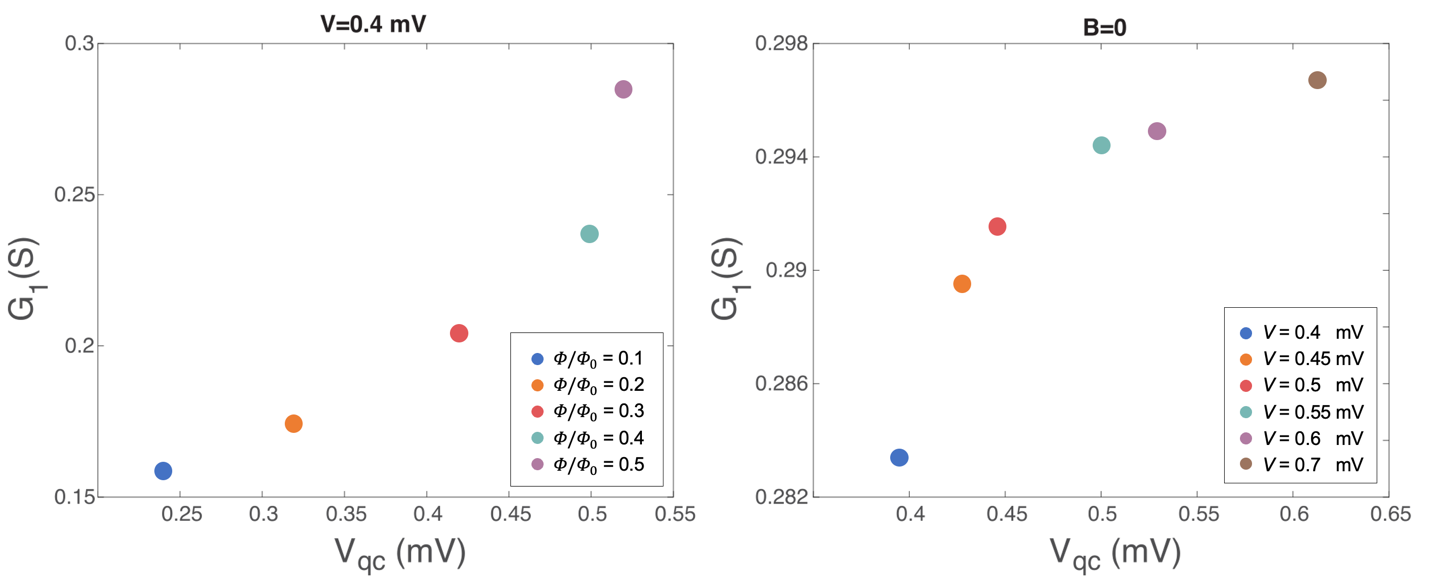


the Fig. Supplementary 5. Experimentally, the quartet conductance $G_{1}$ along the $V_{1}=-V_{2}=V$ is indeed found to increase monotonically as $V_{qc}$ increases at a fixed bias voltage (V=0.4 mV) with varying magnetic flux (left panel: the five data points correspond to $\Phi/\Phi_{0}$=0.1, 0.2, 0.3, 0.4, 0.5, left to right, respectively). Same monotonic relation between the quartet conductance and quartet critical current for a fixed magnetic field with varying bias voltage $V$ is shown in the right panel (the six data points correspond to *V*=0.4, 0.45, 0.5, 0.55, 0.6, 0.7 mV, respectively).

1. **Flux periodicity of the conductance**

The present device contains a loop in the "quartet source terminal" $S_{0}$. The resulting interferences demonstrate the phase coherence of the DC quartet mode, despite the presence of biases. Moreover, they exhibit oscillations with period $\Phi_{0}/2=hc/4e$ instead of $\Phi_{0}$. As explained in the main text, the ${\Phi_{0}}/2$ period is the fundamental one, it signals the mutual interference of quartets emitted by distinct branches, revealing their charge $4e$. And the $\Phi_{0}$ period is *subharmonic* due to the possibility of splitting the quartets. The flux indeed directly affects the quartet process and it can be used as a probe of the nontrivial effect of the voltage. This is essential for the interpretation of the non-monotonous $G(V)$ variation found in Fig. 4 (main text). Notice the counterintuitive "inversion" found in $I_{c}(\Phi)$ as $V$ increases (namely $I_{qc}(\Phi=0)<I_{qc}\left( \Phi=\frac{\Phi_{0}}{2} \right)$ if the normal region is in the short junction limit [35].

.


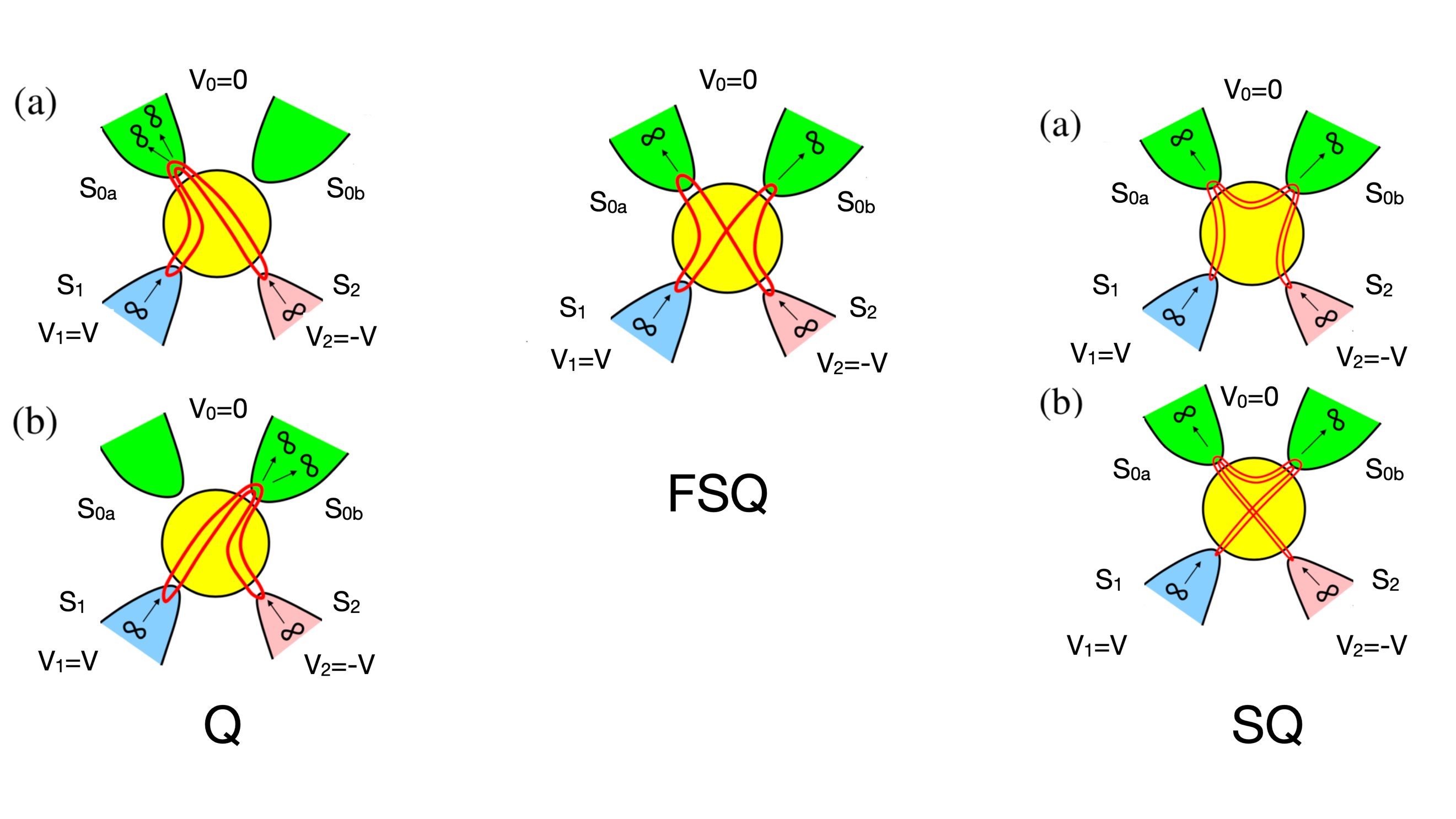


Fig. Supplementary 6. Representation of the lowest order quartet processes. (Left) Q-processes. (Middle) FSQ-process, a split quartet process that vanishes by statistical fluctuations in a multichannel diffusive junction. (Right) SQ-processes.

The meaning of such an inversion is revealed by the perturbative diagrams shown on Fig. Supplementary 6. Due to the high transparency of the junction, this is not quantitative but it illustrates the origin of the flux periodicities. The above diagrams can further be dressed by higher-order lines. The maximum number of lines representing propagating amplitudes is determined by the transparency of the Graphene-Aluminum interfaces. First, the quartet "source" which is the grounded terminal can be either $S_{0a}$ or $S_{0b}$. In this case, both pairs forming the quartet emerge from the same branch $S_{0a}$ or $S_{0b}$, which we denote as Q processes. Alternatively, one pair can emerge from $S_{0a}$ and one from $S_{0b}$. The diagram in the center of Figure Supplementary 6-1 corresponds to "splitting" the quartet in two entangled processes due to the exchange of two fermions [28, 31]. We call this diagram FSQ (Fluctuating Split Quartet) because in a multichannel junction, due to spatial phase fluctuations, the dominant split quartet diagram that survives disorder has two more propagating lines (SQ) and involves the exchange of a quasiparticle between terminals $S_{0a}$_,_ $S_{0b}$.

The two Q-processes differ by the phase acquired by four electrons instead of two for an usual superconducting loop, yielding a periodicity $\Phi_{0}/2=hc/4e$, whatever the interface transparencies at the contacts. In terms of the superconducting phase variables, going from Q diagrams to SQ (FSQ) diagrams formally transfers one pair of the quartet $S_{0a}$ (resp. $S_{0b}$) to $S_{0b}$ (resp. $S_{0a}$), which implies a phase change $2\pi\Phi/\Phi_{0}$. Therefore, the *interference* between the Q and the SQ (FSQ) processes creates a periodicity $\Phi_{0}$ that reminds the one of an ordinary SQUID.

This lowest order calculation is very useful to show how the inversion of the flux dependence can occur. Expliciting the quartet phase, one can write the sum of the Q, SQ (or FSQ) processes as:

$I(\varphi_{q},\Phi)=I_{c,Qa}sin(\varphi_{q}-\Phi)+I_{c,Qb}sin(\varphi_{q}+\Phi)+I_{SQ}sin\varphi_{q}.$

Here the prefactors can take positive or negative values. Quartets are generically π-junctions in the tunnel regime and at vanishing voltage, *i.e.*, $I_{c,Qi}<0$, (i=a, b). Taking the maximum with respect to $\varphi_{q}$ of $I(\varphi_{q},\Phi)$ yields the quartet critical current. For $\Phi=0$ one has $I_{qc}(0)=|I_{c,Qa}+I_{c,Qb}+I_{SQ}|$ while for $\Phi=\Phi_{0}/2$ one has $I_{qc}(\Phi_{0}/2)=|I_{c,Qa}+I_{c,Qb}-I_{SQ}|$*.* The relative sign of $I_{c,Qa,b}$ and $I_{SQ}$ thus governs the flux variation of $I_{qc}$. If it is positive, the total quartet critical current has a maximum at $\Phi=0$ and a minimum at $\Phi=\Phi_{0}/2$, *i.e.*$I_{qc}(\Phi=0)>I_{qc}(\Phi=\Phi_{0}/2)$. If it is negative, the minima and maxima are inverted and $I_{qc}(\Phi=0)<I_{qc}(\Phi=\Phi_{0}/2)$. Importantly, a pure ${\Phi_{0}}/2$ periodicity implies that the SQ channel is absent, leaving only the Q contributions. In this perturbative and short junction limits, calculations show that an inversion can indeed be found [28, 32]. The next section explains more generally, in a nonperturbative dot model, how the new control variable $V$ can indeed trigger an inversion in the sign of $I_{qc}(\Phi=0)-I_{qc}(\Phi=\Phi_{0}/2)$.

1. **The dot model, numerical results and the Floquet-Landau-Zener interpretation**

**a. The single channel dot model.**

This dot model [33] treats the metallic junction as a 0D object with a single level having one-electron energy ${\epsilon_{0}}/2$, no Coulomb interaction and coupled to the four terminals by one-electron matrix elements $t_{i}e^{i\varphi_{i}/2}$. Here i=(1, 2, 0a, 0b) and the superconducting phases are incorporated in the matrix elements. With a convenient choice of the gauge, the phases $\varphi_{i}$ are

$\varphi_{1}=[\varphi_{q}+\varphi_{r}(t)]/2$, $\varphi_{2}=[\varphi_{q}-\varphi_{r}(t)]/2$, $\varphi_{0a}=\Phi/2$, $\varphi_{0b}=-\Phi/2$,

with $\varphi_{r}(t)=4eVt/\hbar$.

The Hamiltonian reads:

$$H_{dot}(t)=\underset{ik\sigma}{\sum}\epsilon_{ik\sigma}c_{ik\sigma}^{\dagger}c_{ik\sigma}+\underset{ik}{\sum}\Delta(c_{ik\uparrow}^{\dagger}c_{i,-k\downarrow}^{\dagger}+H.c.)+\underset{ik\sigma}{\sum}[t_{i}e^{i\varphi_{i}(t)/2}c_{ik\sigma}^{\dagger}d_{i\sigma}+H.c.]+\epsilon_{0}\underset{\sigma}{\sum}d_{\sigma}^{\dagger}d_{\sigma}.$$

The dot energy $\epsilon_{0}$ mimics the control of the metallic junction by a gate.

The model can further be simplified in the large gap limit $\Delta\gg\epsilon_{0},t_{i}$. Single particle processes are traced out and only pair processes remain:

$H_{dot,\infty}(t)=\epsilon_{0}(2b^{\dagger}b-1)+\Gamma(t)b+\Gamma^{*}(t)b^{\dagger}$

with $b=d_{\downarrow}d_{\uparrow}$ and $\Gamma(t)=\Gamma_{0a}e^{i\Phi/2}+\Gamma_{0b}e^{-i\Phi/2}+\Gamma_{1}e^{i[\varphi_{q}+\varphi_{r}(t)]/2}+\Gamma_{2}e^{i[\varphi_{q}-\varphi_{r}(t)]/2}$

with $\Gamma_{i}\sim\pi t_{i}^{2}N(0)$ where $N(0)$ is the Aluminum density of states. The reduced Hamiltonian $H_{dot,\infty}(t)$ describes a driven two-level system and it can be solved with Floquet techniques. In fact the Hamiltonians $H_{dot}(t)$ and $H_{dot,\infty}(t)$ describe a system periodically driven by the running phase $\varphi_{r}(t)=4eVt/\hbar$. This can be compared to driving a Josephson junction by a microwave field, with an important difference: here the drive amplitude is given by $\Gamma_{1},\Gamma_{2}$ and it is *non-perturbative*. Yet, there are similarities: an adiabatic regime holds when the drive frequency is much smaller than the equilibrium Andreev gap. And when it is comparable, Landau-Zener-like transitions occur that couple non-perturbatively the two Andreev levels [37].

Let us comment on the effect of the flux in the large gap limit. $\Gamma(t)$ can be rewritten as $\Gamma(t)=\Gamma_{0}(\Phi)e^{i\alpha(\Phi)}+\Gamma_{1}e^{i[\varphi_{q}+\varphi_{r}(t)]/2}+\Gamma_{2}e^{i[\varphi_{q}-\varphi_{r}(t)]/2}$

with $\Gamma_{0}(\Phi)=\sqrt{\Gamma_{0a}^{2}+\Gamma_{0b}^{2}+2\Gamma_{0a}\Gamma_{0b}cos\Phi}$ , $Tan[\alpha(\Phi)]=\frac{\Gamma_{0a}-\Gamma_{0b}}{\Gamma_{0a}+\Gamma_{0b}}Tan[\Phi/2].$

Therefore, in this limit, the role of the loop is to map the four-terminal junction onto a three-terminal one with a *flux-dependent* coupling to S_c_, together with a phase shift $\alpha(\Phi)$ that can be absorbed in the definition of the quartet phase $\varphi_{q}$. Nevertheless, the dependence of the quartet dynamics on the coupling $\Gamma_{c}$ is far from trivial, it is non-monotonous and thus the flux knob reveals the non-adiabatic effects that explain the experimental observation.

**b. The adiabatic solution.**
At a given time t, the instantaneous ABS energies are given by $E_{ABS}(t)=\pm\sqrt{\epsilon_{0}^{2}+|\Gamma(t)|^{2}}$. In the limit $eV\ll(\epsilon_{0},\Gamma_{i})\ll\Delta$, one can average out the slow drift motion of the phase. $\varphi_{r}(t)$ *i.e.* on the period $T=h/2eV$ to yield adiabatic ABSs, that only depend on the quartet phase $\varphi_{q}$ and on the flux $\Phi$:

$\langle E_{ABS}\rangle(\varphi_{q},\Phi)=\pm\frac{1}{T}\int_{0}^{T}\sqrt{\epsilon_{0}^{2}+|\Gamma(t)|^{2}}dt.$

Importantly, these adiabatic ABS do not depend on the voltage $V$. They give rise to an adiabatic quartet current $I_{q,adiab}=(2e/\hbar)\partial\langle E_{ABS}\rangle/\partial\varphi_{q}$.

In the related case of a microwave-irradiated junction with frequency $\omega$, the adiabatic approximation is controlled both by the smallness of the parameter $\hbar\omega/\delta_{ABS}$ (where $\delta_{ABS}$ is the ABS mini gap at equilibrium) and by the small amplitude of the microwave field. Remind that in our case, even if $\delta_{ABS}$ is not zero and $eV/\delta_{ABS}$ is small, the periodic drive is strong.

c**. Numerical solution of the single-channel dot model.**

As $V$ increases in the experiment, the conductance oscillation has main period $\Phi_{0}$, then its frequency doubles with period ${\Phi_{0}}/2$, and at higher $V$ it recovers the period $\Phi_{0}$ but with a phase shift equal to $\pi$. The frequency doubling corresponds to vanishing of the SQ (FSQ) processes, leaving nearly pure quartet Q_a_, Q_b_ processes interfering. Therefore, at a crossover voltage there is a change of the relative signs of the Q and SQ (FSQ) components.

This scenario is supported by numerical results obtained at nonzero voltage with the dot model and a finite superconducting gap. We use Keldysh non-equilibrium Green’s functions to solve the dynamics of the model described by Hamiltonian $H_{dot}(t)$ [36]. The quartet current-phase characteristics is obtained for any $V$ and $\Phi$. Typical parameters are $\epsilon_{0}=0$ (resonant dot) and:

$\Gamma_{1}=0.4\Delta,\Gamma_{2}=0.2\Delta,\Gamma_{0a}=(0.3\Delta+\gamma)/2,\Gamma_{0b}=(0.9\Delta+\gamma)/2,$

where $\gamma$ controls the relative strength of the $S_{0}$ coupling relatively to the $S_{1},S_{2}$ couplings. More generally, to obtain a $\pi$-shift of the $I_{qc}(\Phi)$ oscillation one needs: (i) a minimum asymmetry $\frac{|\Gamma_{0a}-\Gamma_{0b}|}{\Gamma_{0a}+\Gamma_{0b}}$ of the contacts $S_{0a}$, $S_{0b}$, (ii) a minimum ratio $\frac{\Gamma_{0a}+\Gamma_{0b}}{\Gamma_{1}+\Gamma_{2}}$, (iii) small enough $\epsilon_{0}$.

Figure Supplementary 7 shows the quartet critical current as a function of $V$, for both $\Phi=0$ and $\Phi=\Phi_{0}/2$ (with $\gamma=0.3$). It contains two inversion regions, the main one being at higher voltage. Other narrower inversions are also observed at lower voltages (not represented here).

Fig. Supplementary 7. (Left) Maximum quartet current for $\Phi=0$ (black) and $\Phi=\Phi_{0}/2$ (red), showing two "inversion" windows. (Right, bottom) Same but on an extended voltage range. (Right, top) The corresponding Andreev-Floquet ladders, showing the correlation of the anticrossings with the minima of $I_{qc}$.


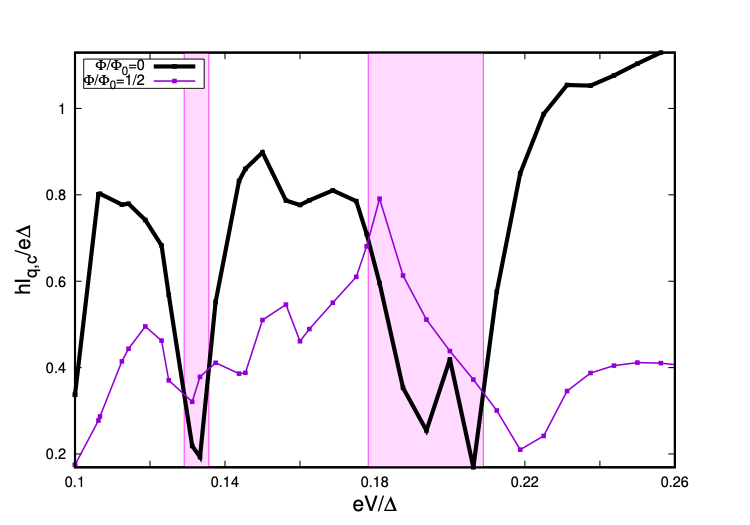

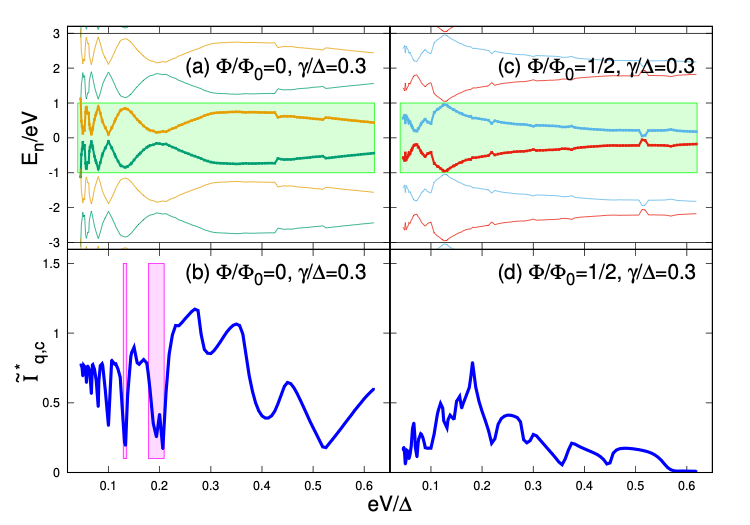


d**. The Floquet ladders and the Rabi (*Landau-Zener*) resonance.**

In this model, the inversion relies on the periodic drive forming Floquet bands from the initial equilibrium ABS. The adiabatic energies $\langle E_{ABS}\rangle$ allow to form Floquet ladders. Here the quartet phase plays the same role as a one-dimensional crystal momentum and the ladders are similar to Wannier-Stark ladders [33]. The Floquet ladders replicate $\langle E_{ABS}\rangle$, following the "classical" formula:

$E_{n,\pm,class}(\varphi_{q},\Phi,V)=\pm\langle E_{ABS}\rangle(\varphi_{q},\Phi)+2neV.$

This picture is correct as far as the Floquet bands are far from each other, i.e. when their spacing $2eV$ is much larger than their dispersion $\sim\Gamma$. Decreasing $V$, when two Floquet bands $E_{n,+,class}$ and $E_{m,-,class}$ cross each other, an avoided crossing happens. This corresponds to a resonance of first order if $|n-m|=1$. Due to the non-perturbative character of the periodic drive, each Floquet band is affected by the presence of its neighbors or of others (higher-order resonances). Anticrossings are due to Landau-Zener-Stückelberg transitions in the instantaneous ABS spectrum $E_{ABS}(t)$: as the running phase slowly drifts, non-adiabatic transitions are obtained close to the minima of the ABS gap $\sqrt{\epsilon_{0}^{2}+|\Gamma(t)|^{2}}$, which can even vanish if $\epsilon_{0}=0$.

Anticrossings and deformations of the Floquet bands cause a nonlinear dependence of the spectrum $E_{n,\pm}(\varphi_{q},\Phi,V)$ with the voltage. Let us now relate the inversions of $I_{qc}(\Phi)$ to anticrossings of "classical" Floquet bands.

Figure S7 also shows a part of the Floquet spectrum (inside the superconducting gap) as a function of the reduced voltage $eV/\Delta$, in a more extended voltage range. The plot represents $E_{n,\pm}/eV$. The anticrossings are visible around precise voltage values, and they get more and more frequent at low voltage. The quartet critical current $I_{qc}$ is represented for both $\Phi=0$ and $\Phi=\Phi_{0}/2$. The minima of $I_{qc}$ are perfectly correlated to the resonances. As for a microwave-irradiated junction, the anticrossings manifest a quantum mixing of the adiabatic states $(n+,m-)$, that bear opposite indices therefore carry opposite currents. As a consequence, in the region of the anticrossing, quantum fluctuations reduce the quartet current $I_{qc}$.

Let us now consider the flux dependence of the position of these resonances and $I_{qc}$ minima. Roughly speaking, the role of the flux is to modulate the coupling $\Gamma_{c}(\Phi)$. This in turn modifies the adiabatic ABS spectrum, its gap and the rate of Landau-Zener transitions. The position of the resonances on the V-axis therefore oscillates periodically with the flux. Figure Supplementary 7 indeed shows that the minima of $I_{qc}(\Phi=0)$ and those of $I_{qc}(\Phi=\Phi_{0}/2)$ do not coincide. This causes a crossing of the curves representing $I_{qc}(V,\Phi=0)$ and $I_{qc}(V,\Phi=\Phi_{0}/2)$. The phenomenon is quite robust against a variation of the couplings $\Gamma_{i}$.

One sees on Figure Supplementary 8 that below and above the resonance (top and bottom panels), the quartet characteristics is a $\pi$-junction, with essentially the period $\Phi_{0}$. Close to the inversion point (central panels) it has a strong ${\Phi_{0}}/2$ period component featuring Q processes.


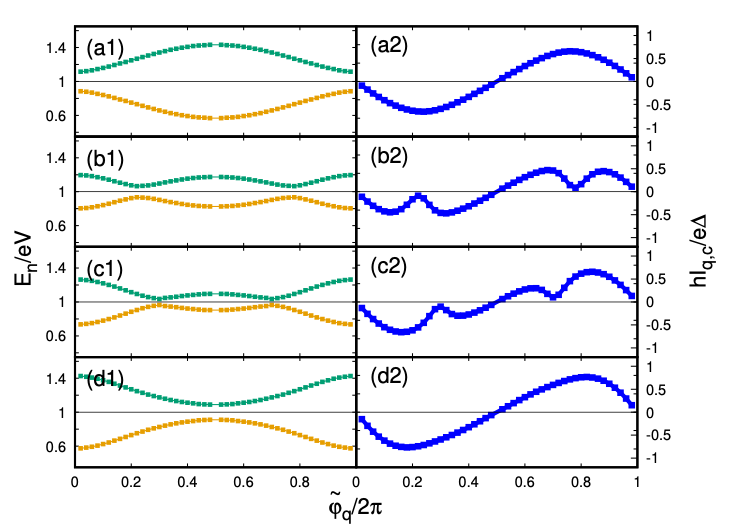


Fig. Supplementary 8. (Right) Evolution of the quartet current-phase characteristics through a Landau-Zener resonance (from a2 to d2). (Left) The corresponding evolution of the Andreev-Floquet bands.

Let us comment on the relevance of the single-channel dot model. First, it requires some asymmetry of the junction contact, calling for a more general scenario for a symmetric junction. Second, it does yield the inversion at very low voltage, due to high-order resonances, but only in narrow voltage ranges. Third, a more realistic model should take into account many channels in the junction. For those reasons, we now consider a two-channel model as a simple approach to future multichannel configurations.

Fig. Supplementary 9 : Sketch of a two-channel model, carried by two quantum dots in parallel, with "direct" and "crossed" couplings $\Gamma$, $\Gamma^{'}$, and interdot coupling $\Sigma$.
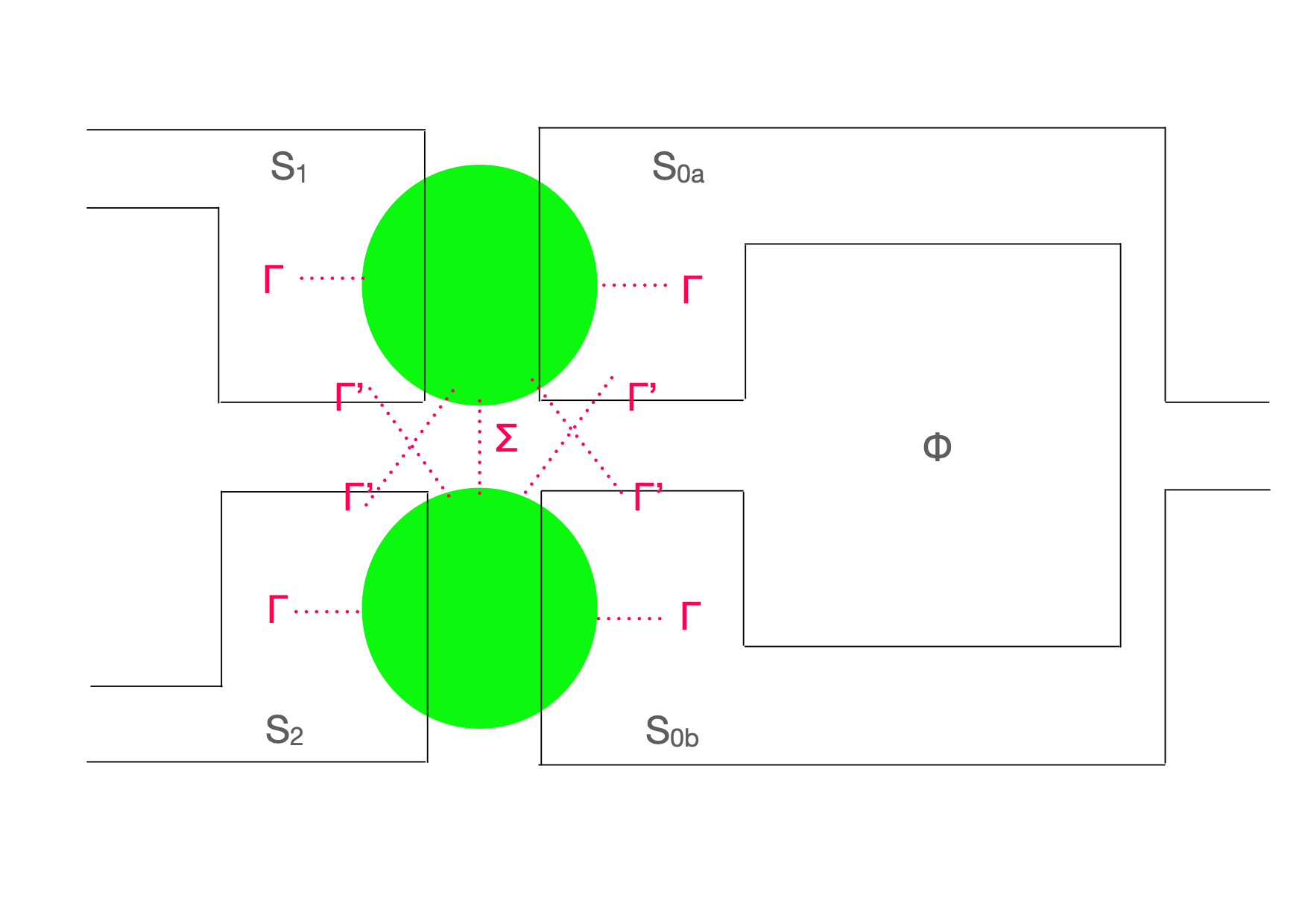


e**. A two-channel dot model**

We show here that the simplest extension of the previous model removes the above mentioned drawbacks, and paves the way for multichannel models that can only be solved numerically. Let us consider a double dot (DA-DB) in parallel (with respect to the loop branches), with "direct" couplings $\Gamma$ and "crossed" couplings $\Gamma^{'}$, and a mutual coupling $\Sigma$ (see Figure Supplementary 9). Importantly, $\Sigma$ mimics the effect of the underlying graphene layer, and calculations allow to take $\Sigma$ complex. The dots are taken here to be resonant (*i.e.* having zero level energies). In the infinite-gap limit, this system is described by the Hamiltonian:

$H_{DD}=\left( \begin{matrix} \varepsilon_{A} & \gamma_{A} & \Sigma& 0 \\ \gamma_{A}* & -\varepsilon_{A} & 0 & -\Sigma\\ \Sigma* & 0 & \varepsilon_{B} & \gamma_{B} \\ 0 & -\Sigma* & \gamma_{B}* & -\varepsilon_{B} \end{matrix} \right)$

with $\gamma_{A}=\Gamma(e^{i\varphi_{1}}+1)+\Gamma^{'}(e^{i\varphi_{2}}+e^{i\Phi/\Phi_{0}})$ and $\gamma_{B}=\Gamma^{'}(e^{i\varphi_{1}}+1)+\Gamma(e^{i\varphi_{2}}+e^{i\Phi/\Phi_{0}})$. Contrary to the single-channel model, even in a symmetric junction, this model allows to find inversion in the flux dependence in the **"**$V=0^{+}$**"**  limit, i.e. of $I_{q,adiab}=(2e/\hbar)\partial\langle E_{ABS}\rangle/\partial\varphi_{q}$ where $E_{ABS}$ is the sum of the two low-lying ABS level energies. This is favored by a ratio $\frac{\Gamma}{\Gamma^{'}}>1$ and by a sizeable interdot coupling $\Sigma$. This can be understood as an effect of interference between the various quartet channels labeled above as "direct"  (Qa, Qb) and "split quartet", together with the two available channels in the junction.

As a consequence, as shown by numerical calculations based on Keldysh Green’s functions, a robust inversion can occur at very low voltage, in a sizable voltage range. Figure Supplementary 10 shows three examples for a symmetric junction, with an intermediate direct coupling, taken as $\Gamma=\Delta$. In the left panel, with no crossed coupling $\Gamma^{'}$ and a real interdot coupling, inversion occurs but at high voltage. In the central panel, the small imaginary part of $\Sigma$ strongly favors a wide and robust inversion at low voltage, as found in the experiment. This is a significant feature : it mimics an hybridization of the two dot channels with an underlying continuum density of states, that provides available states at energies $(E,-E)$ and favors the quartet channel. Last, in the right panel, the effect of an indirect coupling can interchange the order of $I_{qc}(\Phi=0)$ and $I_{qc}(\Phi=\Phi_{0}/2)$. More investigations are required to fully understand the mechanism driving the non monotonous variation of the quartet current with $V_{Q}$ at low voltages, compared to that explained above in the single-channel model at higher voltages.

A numerical study of a many-level dot model was also carried out in Ref. 29, in a wide range of parameters so as to mimic a multichannel junction. Generically, regions of inversion are also found as the voltage is varied, showing the robustness of the phenomenon. Finally, preliminary calculations indicate that in a more general picture of a metallic junction, a more general reasoning based on Keldysh Green’s function also gives rise to inversions (see [32] for more details).
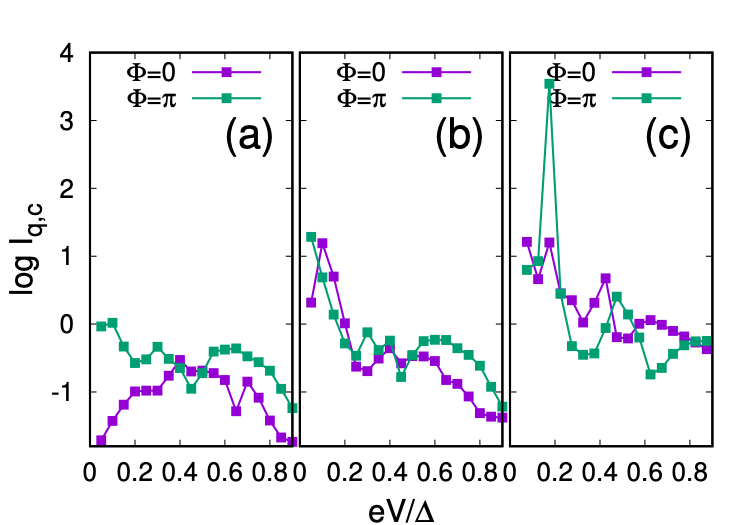


Fig. Supplementary 10. Maximal quartet current in the two-channel model, as a function of the quartet voltage $V=V_{Q}$. Direct coupling $\Gamma=\Delta$, real part of the interdot coupling $Re(\Sigma)=0.2\Delta$, and (Left) crossed coupling $\Gamma^{'}=0$, imaginary part of the interdot coupling $Im(\Sigma)=0$; (Middle) $\Gamma^{'}=0$, $Im(\Sigma)=0.05\Delta$; (Right) $\Gamma^{'}=0.5\Delta$, $Im(\Sigma)=0.05\Delta$..

**Section 6. Critical current contour oscillations**

In Fig. Supplementary 11 we measure differential conductance of the multi-terminal JJ as a function of magnetic field and along the *V_1_=V_2_* condition. In this measurement we are performing a cut along the +45° line of the critical current contour and observing its behavior as a function of magnetic field. This is an important point considering previous works on MT-JJ, specifically Ref [11] in our main text.

**
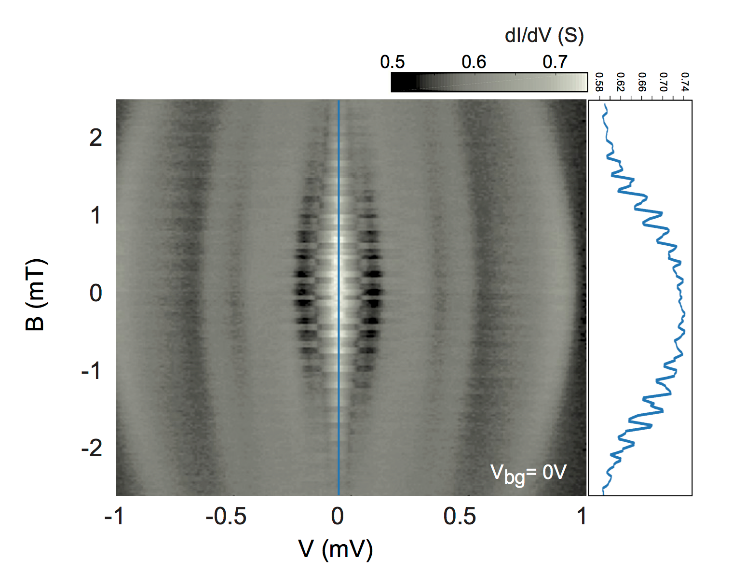
**Fig. Supplementary 11: Magnetic field dependence, $dI/dV$ as a function of the bias voltage and magnetic field at $V_{bg}$= 0V. Bright region (high conductance) is the supercurrent, and the edge corresponds to the value of critical current, which is modulated by the magnetic field. The SQUID-like pattern indicates the critical current contour oscillations.

**Section 7. Phase evolution along an incommensurate bias condition**

In Fig. Supplementary 12 we present the normalized differential conductance of the multi-terminal JJ as a function of magnetic field and V_1_ at a fixed V_2_.


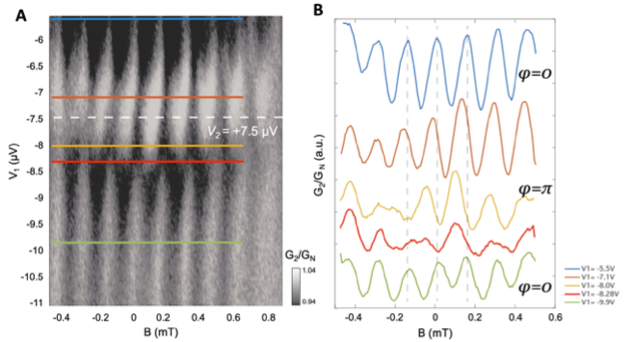


Fig. Supplementary 12: **A.** G_2_/G_N_ as a function of magnetic fields and *V_1_* at fixed *V_2_*=+7.5 mV. **B.** Traces obtained from **A**, along fixed *V_1_* to demonstrate 0-π phase change only occurs near *V_1_=-V_2_*.

References

[1] J.-D. Pillet, C. H. L. Quay, P. Morfin, C. Bena, A. L.

Yeyati, and P. Joyez, Nat. Phys. 6, 965 (2010).

[2] W. Chang, V. Manucharyan, T. S. Jespersen, J. Nygård, and C. M. Marcus, Phys. Rev. Lett. 110, 217005 (2013).

[3] L. Bretheau et al., Nat. Phys.13, 756 (2017).

[4] L. Bretheau, Ç. Ö. Girit, H. Pothier, D. Esteve, and C. Urbina, Nature 499, 312 (2013).

[5] D.J. van Woerkom et al., Nature Physics 13, 876 (2017).

[6] A. Barone, & G. Paternò, Physics and Applications of the Josephson Effect. Physics and Applications of the Josephson Effect (Wiley, 1982).

[7] T. M. Klapwijk, G. E. Blonder, and M. Tinkham, Physica (Amsterdam) 109-110B, 1657 (1982); M. Octavio, M. Tinkham, G. E. Blonder, and T. M. Klapwijk,

Phys. Rev. B 27, 6739 (1983).

[8] D. Averin and A. Bardas, Phys. Rev. Lett. 75, 1831 (1995).

[9] E. N. Bratus’, V. S. Shumeiko, and G. Wendin, Phys. Rev. Lett. 74, 2110 (1995).

[10] J. C. Cuevas, A. Mart\' in-Rodero and A. Levy Yeyati, Phys. Rev. B 54, 7366 (1996).

[11] M. Chauvin et al., Phys. Rev. Lett. 99, 067008 (2007).

[12] C. Byers and T. Flatté, Phys. Rev. Lett. 74, 306 (1995).

[13] J. Torrès, & T. Martin, Eur. Phys. J. B 12, 319–322 (1999).

[14] G. Deutscher and D. Feinberg, Appl. Phys. Lett. 76, 487 (2000).

[15] M. S. Choi, C. Bruder, & D. Loss, Phys. Rev. B 62, 13569 (2000).

[16] G.Falci,D.Feinberg,and F.W.Hekking,Europhys.Lett.54,255 (2001)

[17] G. B. Lesovik, T. Martin, & G. Blatter, Eur. Phys. J. B 24, 287–290 (2001).

[18] D. Beckmann, H. B. Weber, & H. V. Lohneysen, Phys. Rev. Lett. 93, 197003 (2004).

[19] S. Russo, M. Kroug, T. M. Klapwijk, & A. F. Morpurgo, Phys. Rev. Lett. 95, 027002 (2005).

[20] P. C. Zimansky, & V. Chandrasekhar, Phys. Rev. Lett. 97, 237003 (2006).

[21] L. G. Herrmann, F. Portier, P. Roche, A. L. Yeyati,

T. Kontos, and C. Strunk, Phys. Rev. Lett. 104, 026801 (2010).

[22] L. Hofstetter et al., Nature 461, 960 (2009).

[23] A. Das, Y. Ronen, M. Heiblum, D. Mahalu, A. V. Kretinin,

and H. Shtrikman, Nat. Commun. 3, 1165 (2012).

[24] Z. B. Tan, D. Cox, T. Nieminen, P. Lähteenmäki, D. Golubev, G. B. Lesovik, and P. J. Hakonen, Phys. Rev. Lett. 114, 096602 (2015)

[25] M.A.H. Nerenberg et al., Phys. Rev. B 21, 118 (1980); D.W. Jillie et al., Phys. Rev. B 21, 125 (1980)

[26] A.K. Jain et al, Phys. Reports 109, 309 (1984).

[27] J. C. Cuevas, & H. Pothier, Voltage-induced Shapiro steps in a superconducting multiterminal structure. Phys. Rev. B 75, (2007).

[28] M. P. Nowak, M. Wimmer, A. R. Akhmerov, Suppercurrent carried by nonequilibrium quasiparticles in a multiterminal Josephson junction. Phys. Rev. B 99, 075416 (2019).

[29] A. Ingerman, G. Johansson, V. S. Shumeiko, G. Wendin, Coherent multiple Andreev reflections and current resonances in SNS quantum point contacts. Phys. Rev. B 64, 144504 (2001).

[30] R. Mélin, Inversion in a four-terminal superconducting device on the quartet line: I. Two-dimensional metal and the quartet beam splitter. Phys. Rev. B 102, 245435 (2020).

[31] R. Mélin, & B. Douçot, Inversion in a four terminal superconducting device on the quartet line: II. Quantum dot and Floquet theory. Phys. Rev. B 102, 245436 (2020).

[32] R. Mélin, Phys. Rev. B 105, 155418 (2022)

[33] R. Mélin et al., Phys. Rev. B 95, 085415 (2017); Ibid., Phys. Rev. B 100, 035450 (2019).

[34] Y. Cohen et al., PNAS 115, 6991 (2018).

[35] R. Mélin, Multi-terminal ballistic Josephson junctions coupled to normal leads. Phys. Rev. B 105, 155418 (2022).

[36] R. Mélin et al., Phys. Rev. B 93, 115436 (2016).

[37] F. S. Bergeret et al. Phys. Rev. Lett. 105, 117001 (2010).
